# Non-invasive monitoring of intracranial pressure changes: healthy volunteers study

**DOI:** 10.3389/fphys.2023.1208010

**Published:** 2023-08-08

**Authors:** Maria Roldan, George R. E. Bradley, Elisa Mejía-Mejía, Tomas Y. Abay, Panayiotis A. Kyriacou

**Affiliations:** Research Centre for Biomedical Engineering, City University of London, London, United Kingdom

**Keywords:** intracranial pressure, photoplethysmography, near infrared spectroscopy, brain monitoring, machine learning

## Abstract

**Objective:** This research aims to evaluate the possible association between pulsatile near infrared spectroscopic waveform features and induced changes in intracranial pressure in healthy volunteers.

**Methods:** An optical intracranial pressure sensor was attached to the forehead of 16 healthy volunteers. Pulsatile near infrared spectroscopic signals were acquired from the forehead during body position changes and Valsalva manoeuvers. Features were extracted from the pulsatile signals and analyses were carried out to investigate the presence of statistical differences in the features when intracranial pressure changes were induced. Classification models were developed utilizing the features extracted from the pulsatile near-infrared spectroscopic signals to classify between different body positions and Valsalva manoeuvre.

**Results:** The presence of significant differences in the majority of the analyzed features (p < 0.05) indicates the technique’s ability to distinguish between variations in intracranial pressure. Furthermore, the disparities observed in the optical signal features captured by the proximal and distal photodetectors support the hypothesis that alterations in back-scattered light directly correspond to brain-related changes. Further research is required to subtract distal and proximal signals and construct predictive models employing a gold standard measurement for non-invasive, continuous monitoring of intracranial pressure.

**Conclusion:** The study investigated the use of pulsatile near infrared spectroscopic signals to detect changes in intracranial pressure in healthy volunteers. The results revealed significant differences in the features extracted from these signals, demonstrating a correlation with ICP changes induced by positional changes and Valsalva manoeuvre. Classification models were capable of identifying changes in ICP using features from optical signals from the brain, with a sensitivity ranging from 63.07% to 80% and specificity ranging from 60.23% to 70% respectively. These findings underscored the potential of these features to effectively identify alterations in ICP.

**Significance:** The study’s results demonstrate the feasibility of using features extracted from optical signals from the brain to detect changes in ICP induced by positional changes and Valsalva manoeuvre in healthy volunteers. This represents a first step towards the non-invasive monitoring of intracranial pressure.

## 1 Introduction

Intracranial pressure (ICP) is frequently impaired in neurocritical care patients. There are several conditions which can cause intracranial hypertension, such as, head injury, cerebral haemorrhages, stroke, intracerebral hematomas, meningitis, acute liver failure and hydrocephalus. ICP can also be influenced by surgical interventions such as tumour removal and the repairing of damaged blood vessels (Chiara, 2018). Despite several studies showing that hospital-level ICP monitoring utilisation varies substantially, with some hospitals measuring invasive ICP in as few as 9.5% of their patients, while others monitor up to 83% of their patients (Okazaki et al., 2021; Bennett et al., 2012), it is considered the gold standard in neurocritical monitoring (Evensen and Eide, 2020). Early management of intracranial hypertension decreases the risk of secondary injuries to the brain, poor outcomes and mortality (Chiara, 2018). However, current gold standard monitoring techniques are predominantly measured by invasive methods which rely on neurosurgical expertise, which could potentially delay treatment whilst introducing additional risks for the patient (Roldan et al., 2020). Consequently, numerous authors have searched for non-invasive methods to assess the brain, such as Computerised Tomography Scan, Magnetic Resonance Imaging, Transcranial Doppler and Near-Infrared Spectroscopy (NIRS) (Roldan et al., 2020). The latter has been widely described for cerebral perfusion and brain oxygenation monitoring (Roldan and Kyriacou, 2021). Continuous waveform NIRS and spatially resolved spectroscopy techniques are based on the light absorbance change reflected in the DC component of the optical signal measured by the probe (Roldan and Kyriacou, 2021). However, the information regarding the pulsatile (AC) component of the reflected infrared light has yet to be assessed. This AC component might be associated with changes in intracranial volume, which is highly correlated to ICP. This research aims to evaluate the possible association between pulsatile NIRS waveform features and induced changes in ICP in healthy volunteers.

## 2 Materials and methods

### 2.1 Monitoring device

**FIGURE 1:**
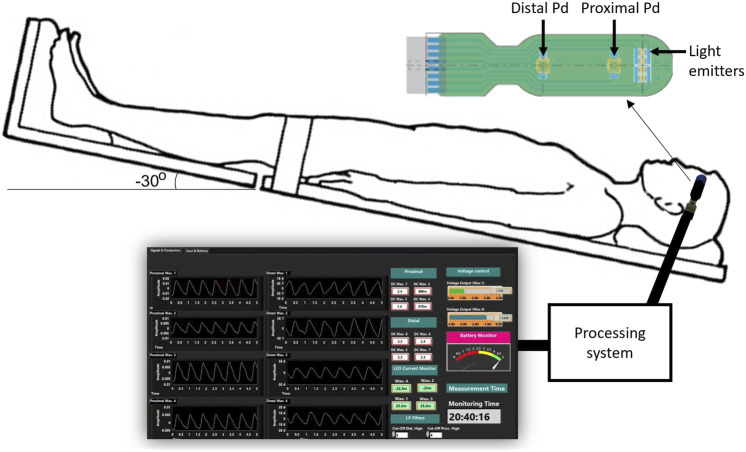
Experimental setup for the acquisition of pulsatile optical signals from the forehead. The sensor has a reflectance configuration with four light emitters (770, 810, 855, and 880 nm). The reflected light from extracerebral tissue reached the proximal photodetetor placed 10 mm from the LEDs. Similarly, non-absorbed light from deeper tissues travelled back to the distal photodiode placed 35 mm from the source. The probe is connected to a processing system to acquire the signals, which are finally visualised and recorded in a LabView interface.

A custom made optical nICP sensor was attached to the subject’s foreheadbelow the hairline. This in-house, non-invasive sensor consists of four LEDs at multiple wavelengths and two photodiodes (proximal and distal) arranged as shown in Figure 1. A multiple wavelength sensor allows for multimodal monitoring, however This study exclusively analyzed data at 810 nm as the absorption properties of oxyhemoglobin and deoxyhemoglobin are the same at this specific wavelength. This characteristic enables the extraction of an optical signal that is independent of blood oxygenation (Murkin and Arango, 2009). Montecarlo simulation of the light-tissue interaction has demonstrated that near-infrared (NIR) light travels deeper into the head tissue when the source-detector distance is increased (Roldan et al., 2021). The reflected light from extracerebral tissue reached the proximal photodetector placed 10 mm from the LEDs; and the non-absorbed light from deeper tissues travelled back to the distal photodiode placed 35 mm from the LEDs. This study interrogated the effect of the photodiodes locations under ICP changes, and evaluated the acquisition of pulsatile signals from both distal and proximal photodiodes. The optical probe was driven by a custom made processing system enabling optical signal acquisition, pre-processing, visualization (using LabView) and archiving on a personal computer.

### 2.2 Healthy subjects

Sixteen healthy volunteers aged 28 ± 6 years (mean ± standard deviation; 6 women) took part in this study. The subjects were recruited through posters in the university where the study took place. The exclusion criteria included existing pathology associated with raised ICP, vasculitis, diabetes, high-risk factor for stroke or heart disease, hypertension, previous traumatic brain injury, meningitis or hydrocephalus, migraine, vertigo, fever, influenza or other infectious diseases. The University Senate Research Ethics Committee approved the study, and all participants signed an informed consent form prior to the study.

### 2.3 Intervention

**FIGURE 2:**
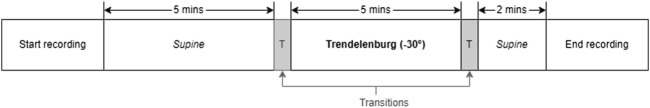
Measurement protocol for the body position tilting intervention.

Literature has established that tilting body position and the Valsalva manoeuver are effective methods of inducing changes in ICP among healthy volunteers (Haykowsky et al., 2002; Eklund et al., 2016; Watkins et al., 2017). In order to replicate these conditions, the volunteers’ body positions were adjusted a tilt-function of the investigation bed. The protocol began with the volunteers in a supine position for 5 min (first supine), followed by a 5-min period in the Tredelenburg position, with the head inclined downward at −30°. The protocol ended with the volunteers returning to a supine position for an additional 2 min (second supine). A transition period of 30–60 s was allowed between each position. During the transition period, data was not recorded. Figure 2 provides a visual summary of this protocol. Similarly, for the Valsalva intervention, volunteers were seated and baseline measurements were recorded before three consecutive Valsalva manoeuvres were performed.

### 2.4 Analysis

The recorded signals were processed and analyzed using Python (version 3.10). The signal processing began by sectioning the recorded signals based on the corresponding body positions. Additionally, the Valsalva peaks were isolated from the baseline. The signals were filtered using Butterworth filters in order to separate the AC PPG component (2nd order bandpass filter with cutoff frequencies of 0.8 and 10 Hz) from the DC PPG component (2nd order lowpass filter with a cutoff frequency of 0.1 Hz). The data was then normalised by dividing the AC component of the signal by it is DC component, followed by a 10 factor multiplication.

### 2.5 Sectioning

**FIGURE 3:**
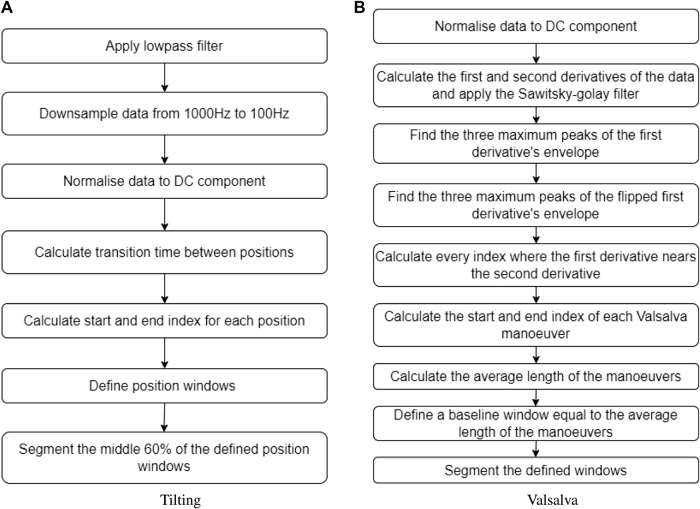
Sectioning algorithms applied to segment data obtained during **(A)** the tilting protocol and **(B)** the Valsalva intervention.

For the tilting intervention, changes in body position were determined by time. The sectioning algorithm under-sampled the signals to 100 Hz and the total duration of the signal was calculated. From the calculated duration, the time spent in transitions was subtracted to obtain an average transition time. Subsequently, the indices corresponding to each body position window were estimated. To ensure that only relevant data from the body position was included and to exclude transition data, the middle 60% of each body position window was extracted. This ensured that the analysis focused on stable body position periods. The flow diagram illustrating this sectioning algorithm can be seen in Figure 3A.

Inversely, the Valsalva manoeuvre peaks were not controlled by time, therefore a different algorithm was required to identify and extract these peaks from the original signal. The first step was calculating the first and second derivative of the PPG signal, which were then filtered using a Savitzky–Golay filter. Then, three maximum peaks were detected from the first derivative envelope, as well as three maximum lows. The algorithm defined where a Valsalva window started when a maximum peak of the first derivative envelope was close to a point where the first and second derivative crossed. Likewise, the Valsalva window ended when a maximum low of the first derivative envelope was close to a point where the first and second derivative crossed. The average time of the three Valsalva peaks was calculated, and a corresponding segment of baseline time was extracted from the signal, representing the period before any Valsalva maneuver occurred. Figure 3B provides a description of the sectioning algorithm used for the Valsalva intervention.

### 2.6 Feature extraction

**FIGURE 4:**
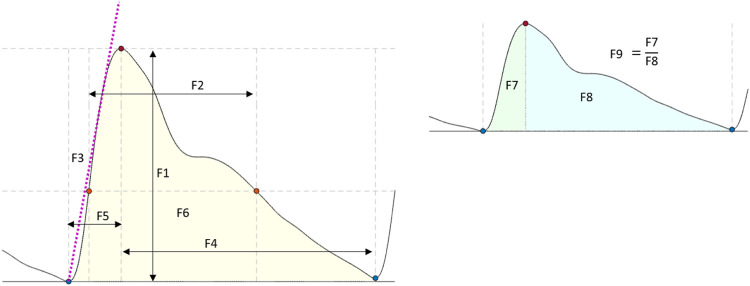
Features extracted from the optical signals. F1: Amplitude; F2: Pulse width; F3: Up slope; F4: Decay time; F5: Rise time; F6: Area under the curve; F7: Area of the systolic period; F8: Area of the diastolic period; F9: Ratio between both systolic and diastolic areas.

Several features have been extracted and investigated in the literature to characterise pulsating signals, such as PPG (El-Hajj and Kyriacou, 2021). In this study, nine features were extracted from the optical signals. These features were: the amplitude, pulse width, rise time, decay time, upslope, area under the curve, area of the systolic period, area of the diastolic period and ratio between both systolic and diastolic areas. Figure 4 shows a graphical representation of the figures extracted. The median value of each feature was calculated in a signal window of 15 s for the tilting intervention while a 5 s window was implemented for the Valsalva intervention, where the sectioned signals were shorter. The features from each section (baseline, body position or Valsalva) were compared according to the following statistical analysis.

### 2.7 Statistical analysis

To assess whether there were any significant differences in the features extracted at different body positions (both proximal and distal measurements), a non-parametric and parametric factorial analysis was undertaken in Python (version 3.10). Utilizing two distinct analytical methods increased the robustness of our investigation and ensured that any significant findings were not reliant upon a particular statistical technique. The significance level was set at 95% (*α* = 0.05) for both analyses. The factors were the body position and the photodetector location.

The ANOVA assumptions were assessed using several statistical tests. Specifically, the normality assumption for each feature was evaluated using the Shapiro-Wilk and Kolmogorov-Smirnov tests, while Bartlett’s test was used to evaluate variance homogeneity for each factor. Data independence was assessed through graphical methods. In instances where the assumptions of normality, homoscedasticity, and independence were not met, Box-Cox transformations were applied using an optimal lambda in order to proceed with the factorial analysis. Following the transformation, both factors were incorporated into a linear model to examine any potential interaction effects on the changes in the extracted features.

Lastly, logisitc regression classification models were constructed to predict body position and Valsalva peaks based on the independent variables extracted from the optical signals recorded with the aforementioned probe. The data underwent pre-processing steps prior to model training. Firstly, all instances except for those at the 810 nm wavelength were removed from the original feature dataset, resulting in a dataset containing only instances of extracted features from the 810 nm wavelength. Secondly, to address the issue of class imbalance, the original dataset was randomly undersampled to ensure an equal number of instances for each classification class (body positions/Valsalva peak or baseline) for each volunteer at each intervention. The independent variables were scaled to a range between 0 and 1. This step was undertaken to help improve the performance and convergence rate of the algorithm, as the features extracted used different ranges. The final pre-processing step for the model involved splitting the tilting and Valsalva datasets by photodetector position (proximal and distal). As distal signals contain cerebral information, the model was trained using the distal photodetector dataset only.

The decision to use a logisitc regression classification model in this study was driven by its suitability for a small labelled dataset and its effectiveness on binary classification tasks. Logistic regression only requires estimation of coefficients associated with each predictor variable, resulting in fewer parameters to estimate compared to more complex models. Consequently, logistic regression is less prone to overfitting. Furthermore, logistic regression takes the form of a sigmoid curve, which ranges between 0 and 1, reflecting the probability of a binary outcome. Therefore, logistic regression is a suitable choice for classification problems where the outcome variable is binary.

To split the data into training and testing datasets, two different approaches were employed. The first approach involved dividing the data into folds, with the number of folds equal to the number of volunteers in the dataset (16 for tilting and 10 for Valsalva). Each fold consisted of all the data from one volunteer. In contrast, the second approach employed a 10-fold cross-validation, where the entire dataset was randomly shuffled and split into 10 folds. In both approaches, the model was trained on the remaining data after holding out the fold and then tested on the held-out data. To evaluate the performance of the models, accuracy measurements, sensitivity and specificity were calculated.

## 3 Results

### 3.1 Pulsatile signals from the forehead

**FIGURE 5:**
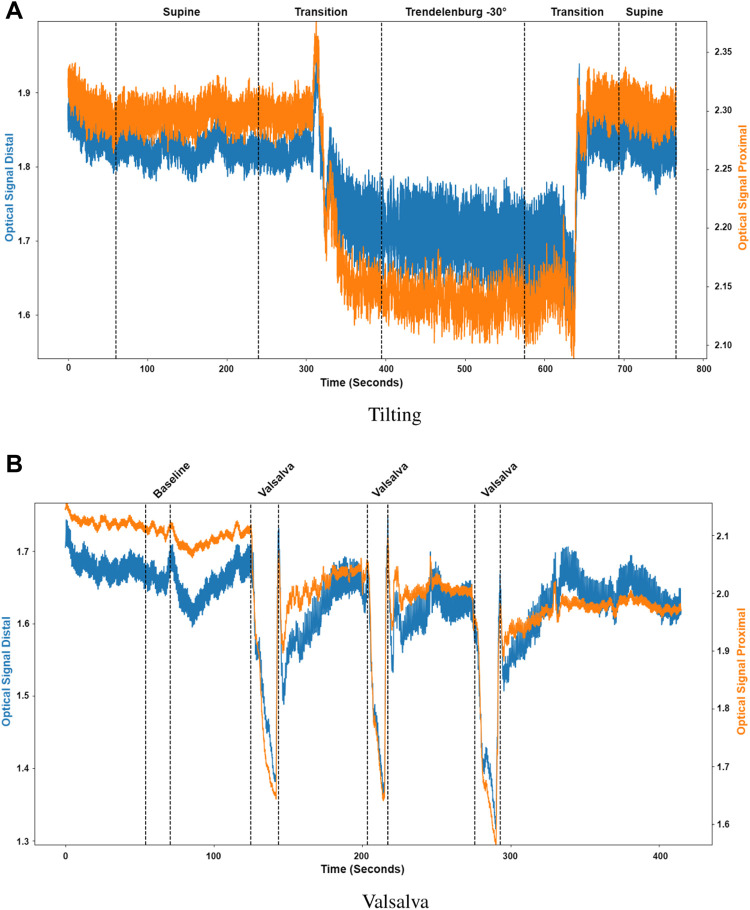
Sectioned signals per intervention. **(A)** Tilting, where the distal (blue) and proximal (orange) signals were sectioned to identify initial supine position, the transition from supine to Trendelenburg (−30°), Trendelenburg position, the transition to supine and final supine position. **(B)** Valsalva, where the distal (blue) and proximal (orange) signals were sectioned to identify the baseline condition and the three different Valsalva maneuvers.

Pulsatile signals were successfully recorded from near infrared light from 16 healthy volunteers using a two photodiode probe. Figure 5 presents the first observational proof of brain pulsatile signals obtained at 810 nm. Additionally, the results of the sectioning algorithm, shown in Figure 5, allow for easy identification of changes in the DC component of the optical signals during ICP alterations (i.e., trendelenburg −30 and Valsalva). Differences in the waveform morphology at different body positions or after a Valsalva manoeuvre, were analysed by feature extraction at each section.

### 3.2 Statistical analysis

**FIGURE 6:**
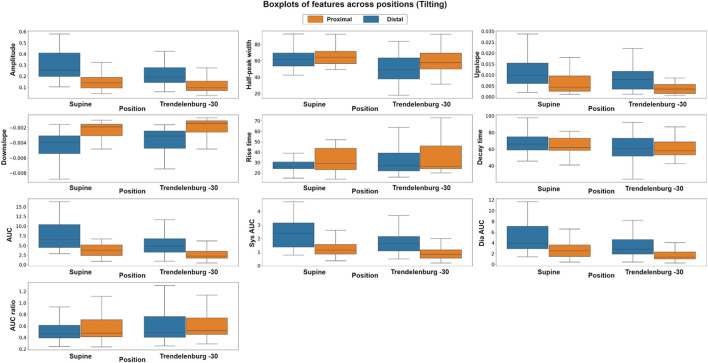
Boxplots of pulsatile signal features at different body positions for both distal and proximal photodiode locations. Each box represents the interquartile range (IQR) of the data, with the median value represented by the horizontal line inside the box. Differences in box height between positions and photodiode locations suggest significant variations in the pulsatile waveform morphology.

To explore the differences in the features extracted at different body positions and between baseline and valsalva manoeuvres, we employed both parametric and non-parametric analyses, the results of which support the hypothesis of this study. Figure 6 shows the boxplots of each feature at the different body positions for both photodiode locations. In the tilting intervention, a non-parametric analysis was conducted using the Mann-Whitney U test. The results indicate significant differences in all features except the rise-time feature. This suggests significant difference between the features across both proximal and distal data.

To complement the non-parametric approach a parametric, factorial analysis using ANOVA was carried out. Although the data did not fit the assumptions of ANOVA in either intervention suggesting that the data were not normally distributed, did not have unequal variances or did not show residual independence a Box-Cox transformation enabled a factorial analysis. After the transformation, the assumptions were evaluated by Q-Q plot, residuals plot and fitted values plot. The results of the ANOVA analysis were consistent with those of the non-parametric analysis. The factorial analysis results indicate significant differences in all features across both proximal and distal measurements. The findings of the comparison between photodiodes showed significant differences between sensor locations across all features, which may be attributed to the fact that the distal photodiode is interrogating mixed brain and extracerebral signals while the proximal photodiode only detects extracerebral signals.

The use of both analytical methods allowed us to confirm our findings using two independent statistical techniques. By performing both parametric and non-parametric analyses, we were able to explore the differences in the features extracted at different body positions producing results which were not reliant upon a particular statistical technique. The evidence of both support our hypothesis that there are significant differences in the features extracted at different body positions.

**FIGURE 7:**
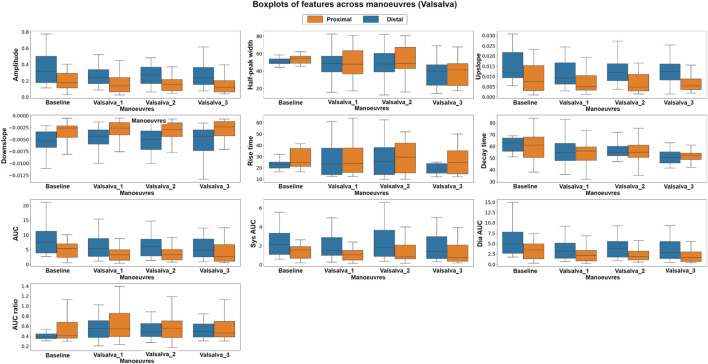
Boxplots of pulsatile signal features at baseline and during three Valsalva manoeuvres for both distal and proximal photodiode locations. Each box represents the interquartile range (IQR) of the data, with the median value represented by the horizontal line inside the box. Differences in box height between baseline and Valsalva conditions, as well as between photodiode locations, indicate significant changes in pulsatile waveform morphology during the Valsalva manoeuvre.

Similarly, in the Valsalva intervention, a non-parametric, Kruskal–Wallis test and a parametric, factorial analysis using ANOVA were conducted to assess the differences between baseline and valsalva for both sensor locations. Due to data quality challenges, data from 10 out of the original 16 volunteers were included in this analysis. Figure 7 presents the boxplots of each feature at baseline and the three Valsalva manoeuvres for both photodiode locations.

The Kruskal–Wallis analysis suggests that the majority of features changed significantly between baseline and Valsalva conditions in the distal dataset, which interrogates mixed brain and extracerebral signals. Conversely, the proximal data revealed that most of the features did not show significant changes between baseline and Valsalva conditions. The ANOVA analysis results were consistent with those of the parametric analysis. The findings indicate significant changes in half of the features analysed between baseline and Valsalva, while the second factor analysis showed significant differences between sensor locations for 70% of the features. These results suggest that the pulsatile signal morphology differs during an instant increase in ICP induced by the Valsalva manoeuvre.

### 3.3 Classification tasks

The results of the classification models are presented on Table 1. Two training and validation approaches were used: the first approach used a hold-out validation method, while the second approach employed a k-fold cross-validation method.

**TABLE 1:** Classification results of hold-out Validation and K-fold cross-validation methods on Tilting and Valsalva Datasets. The classification results include average sensitivity and specificity measures for each approach on each dataset.

Tilting dataset			
Approach	Position	Sensitivity (%)	Specificity (%)
One volunteer per fold	Trendelenburg −30	63.07%	60.23%
K-fold cross validation	Trendelenburg −30	68.1%	63.69%

Valsalva dataset			
Approach	Position	Sensitivity (%)	Specificity (%)
One volunteer per fold	Valsalva	70.0%	70.0%
K-fold cross validation	Valsalva	80.0%	70.0%

On the Tilting intervention dataset the results suggest that both approaches achieved a good balance between correctly identifying trendelenburg −30 and supine cases.

The first approach, the hold-out validation method achieved an average sensitivity of 63.07% and an average specificity of 60.23% on the dataset. In contrast, the k-fold cross-validation method demonstrated slightly better performance, with a sensitivity of 68.1% and a specificity of 63.69%. This suggests that the model has a good balance between identifying both positive and negative cases and is effective at identifying patients in the trendelenburg −30 position.

The favourable classification performance of the second approach is also mirrored in the distal Valsalva dataset. The average sensitivity and specificity of the second approach on the Valsalva dataset were 80.0% and 70.0%, respectively, representing a 10% higher sensitivity than that of the first approach.

The results across both datasets demonstrate an encouraging classification performance using both training and validation approaches. The k-fold cross-validation method showed slightly better results than the hold-out validation method.

## 4 Discussion

**FIGURE 8:**
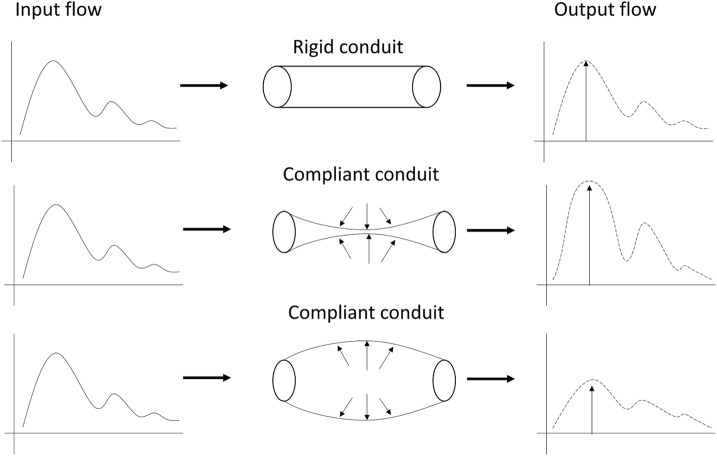
Modulation of pulsatile flow through rigid (top) and compliant (bottom) conduits. Outflow waveform from the compliant conduit suffered changes on the pulse morphology compared to outflow from the rigid conduit. Figure adapted from Alperin et al. (2005), with permission from John Wiley and Sons.

This study has proposed the use of a custom optical sensor with multi-distance photodetectors to interrogate pulsatile signals from backscattered light from the brain. The sensor demonstrated its capability to acquire pulsatile signals from extracerebral and cerebral tissue at multiple wavelengths, of which the isosbestic point (810 nm) was selected for further analysis. Volunteers underwent two different interventions, tilting body position and Valsalva manoeuvre, in order to induce changes in the intracranial pressure. Trendelenburg at −30°, lead to the engorgement of the brain, where blood and Cerebrospinal Fluid (CSF) volume increase the total intracranial volume, hence the ICP. Intracranial hypertension decreases the intracranial compliance and results in an enlarged flow pulsatility (Figure 8) (Alperin et al., 2005). The Results from this study demonstrate that both scenarios of enlargement and attenuation of the flow pulsatility due to changes in ICP, correlate to changes in the morphological features of the pulsatile optical signals in healthy volunteers. Similarly, significant changes in the features of the pulsatile signals during Valsalva manoeuvres represent a rise in the intracranial pressure due to the sudden expulsion of blood from the thoracic vessels into the carotid vessels (Prabhakar et al., 2007).

To the knowledge of the authors, there are no previous studies that have analyzed the features of pulsatile optical signals to discriminate changes in ICP induced by changes in body position and Valsalva manoeuvers. Literature reports significant changes in ICP when healthy volunteers undergo tilting or Valsalva manoeuvres. Alperin et al. quantified the effect of posture on intracranial physiology using Magnetic Resonance Images (MRI) of the brain, demonstrating that changes in ICP induced by posture alterations strongly affect the dynamics of cerebral blood and CSF flows (El-Hajj and Kyriacou, 2021). Computer tomography scans and MRI are non-invasive technologies used for TBI monitoring; however their accessibility is limited and do not facilitate bedside or continuous monitoring. On the other hand, invasive techniques such as intracranial bolts or neuroendoscopic procedures have also been used to evaluate changes in ICP during Valsalva manoeuvres, showing a significant increase in cerebral dynamics, including intracranial hypertension after the intervention (Alperin et al., 2005). Invasive ICP monitoring is an invasive and expensive procedure which requires a high level of expertise. Increasing the barrier to entry for patients and healthcare systems.

Since the analysis of features of pulsatile optical signals was capable of discriminating changes in ICP in healthy volunteers, this method might be of significant value for the future development of prediction algorithms for non-invasive monitoring of absolute ICP values. From the models developed in this study, a conclusion regarding the hold-out method is that it allows for an accurate measure of the model’s performance on each volunteer whilst also giving a representation of the model’s performance on unseen data. However, it seems reasonable to suggest that with further research and the collection of a larger dataset we could expect better model performance from approach one. On the other hand, the K-fold classification is a good method of evaluating a model on a limited dataset and provides a good estimate of the model’s performance on unseen data.

This study has demonstrated that the morphologies of the signals recorded from the sensor differ between baseline and Valsalva conditions as the analysis found extracted features from the signal changed significantly between the two conditions. In addition to this the results found significant differences within features between body positions supine and trendelenburg −30. In order to further suggest that the sensor is isolating signals from the brain the study could be repeated using data recorded from both the forehead and the finger with the intention to measure and evaluate the changes in extracted features from both during baseline and Valsalva conditions and during the protocol of positions. Additionally this study has found meaningful differences within the features of pulsatile NIRS signals which are correlated with induced changes in intracranial pressure. Given this it seems reasonable to explore the capacity to non-invasively measure intracranial pressure using data collected by the sensor. To determine the efficacy of the sensor’s capacity to non-invasively measure intracranial pressure this work could be extended to the simultaneous collection of invasive intracranial pressure data and data from the sensor with the intention to develop a computational approach to calculating intracranial pressure values from the data. The results of which would be measured and compared against the reported gold standard. If it can be demonstrated as being efficacious for the non-invasive measurement of intracranial pressure, the research could be extended to the development of computational approaches for the forecasting of intracranial pressure values at future time points using non-invasively collected data. This would provide a non-invasive and continuous intracranial pressure measurement approach capable of the early detection of intracranial pressure crises.

This study has some limitations. First, and most importantly, gold-standard ICP measurements were not acquired during this study. This was due to the invasive nature of the current ICP monitoring techniques, making it impossible to obtain from healthy volunteers. However, and as has been explained, the effects of the protocols performed in this study have been shown to trigger changes in ICP which was the aim of this study. Future studies should validate the results obtained from this study in critically-ill patients with continuous, invasive ICP monitoring, from which ICP values are available. Secondly, the sample size from this study is relatively small and the characteristics of the subjects were homogeneous, risking overfitting the results of this study to the sample used. Furthermore, only a small subset of features were extracted and analysed from the obtained signals. Future studies should aim to explore more features from pulsatile signals that may be more prone to changes in ICP, such as frequency-domain and nonlinear indices. Additionally only data obtained using light at 810 nm were analysed. This was done due to the importance of this wavelength in the assessment of blood perfusion. However, future studies should evaluate the relationship of signals obtained using other wavelengths to ICP changes. Finally, it has been suggested that the developed sensor acquires pulsatile signals from the extracerebral and cerebral tissues of the brain, from the proximal and distal photodetectors, respectively. This study focused on the distal signal dataset in the classification task, as it is expected to contain cerebral information. However, the proximal data has not been used and the optical subtraction of the data collected from the proximal photodiode from the data acquired from the distal photodiode may isolate the cerebral data by eliminating the noise of extracerebral data, making the results more reliable and resulting in a better performance on the classification task.

## 5 Conclusion

This study found significant differences in the features extracted from the pulsatile NIRS signals, that correlate to induced changes in intracranial pressure in healthy volunteers. Additionlly this study presented classification models capable of identify changes in ICP induced by changes in body position and Valsalva manoeuvres. This novel method might be of significant value for the future implementation of a non-invasive ICP monitoring tool in neurocritical care.

## Data Availability

The raw data supporting the conclusion of this article will be made available by the authors, without undue reservation.
